# Bioactive Neuroelectronic Interfaces

**DOI:** 10.3389/fnins.2019.00269

**Published:** 2019-03-29

**Authors:** Dayo O. Adewole, Mijail D. Serruya, John A. Wolf, D. Kacy Cullen

**Affiliations:** ^1^Center for Brain Injury and Repair, Department of Neurosurgery, Perelman School of Medicine, University of Pennsylvania, Philadelphia, PA, United States; ^2^Department of Bioengineering, School of Engineering and Applied Science, University of Pennsylvania, Philadelphia, PA, United States; ^3^Center for Neurotrauma, Neurodegeneration and Restoration, Corporal Michael J. Crescenz Veterans Affairs Medical Center, Philadelphia, PA, United States; ^4^Department of Neurology, Thomas Jefferson University, Philadelphia, PA, United States

**Keywords:** neurotechnology and brain-machine interface, tissue engineering, biomaterials, neural engineering, neuroprosthetics

## Abstract

Within the neural engineering field, next-generation implantable neuroelectronic interfaces are being developed using biologically-inspired and/or biologically-derived materials to improve upon the stability and functional lifetime of current interfaces. These technologies use biomaterials, bioactive molecules, living cells, or some combination of these, to promote host neuronal survival, reduce the foreign body response, and improve chronic device-tissue integration. This article provides a general overview of the different strategies, milestones, and evolution of bioactive neural interfaces including electrode material properties, biological coatings, and “decoration” with living cells. Another such biohybrid approach developed in our lab uses preformed implantable micro-tissue featuring long-projecting axonal tracts encased within carrier biomaterial micro-columns. These so-called “living electrodes” have been engineered with carefully tailored material, mechanical, and biological properties to enable natural, synaptic based modulation of specific host circuitry while ultimately being under computer control. This article provides an overview of these living electrodes, including design and fabrication, performance attributes, as well as findings to date characterizing *in vitro* and *in vivo* functionality.

## Introduction

Neuroelectronic interfaces, also commonly referred to as neural or brain-computer interfaces, enable the transfer of information between the nervous system and an external device (Hatsopoulos and Donoghue, 2009; Wolpaw, 2013; Adewole et al., 2016). Generally, these devices take the form of electrodes to record or modulate neuronal activity through transducing cellular activity into actionable information (recording) or delivering current into tissue (stimulation) (Cogan, 2008; Grill et al., 2009). Neural interfaces are currently applied in both investigative and clinical contexts, from answering basic neuroscience questions about behavior, information encoding, and mechanisms of injury, to cochlear implants to restore hearing loss, deep brain stimulation to treat Parkinson’s disease, and the direct control of prosthetic limbs or other peripheral devices (Shih et al., 2012; Adewole et al., 2016).

A fundamental design objective for implantable neural interfaces is the maintenance of long-term function *in vivo* (Grill et al., 2009; Harris and Tyler, 2013; Adewole et al., 2016). This article focuses on interfaces for the brain, wherein the dynamic, aqueous environment presents a host of significant obstacles that have, to date, limited the chronic performance of neural interfaces (Harris and Tyler, 2013; Fattahi et al., 2014). The most prevalent of these obstacles may be collectively summarized as a multimodal, sustained foreign body response (FBR) to the implant, which degrades the efficacy of the interface over time (Polikov et al., 2005; Tresco and Winslow, 2011). The FBR has motivated a vast body of research focused on developing electrodes and implant strategies that either address specific elements of the FBR or limit its effects on device performance, with distinct approaches offering discrete improvements. Here we provide a brief overview of the FBR and its implications for neural interface design before exploring strategies for biologically active interfaces, which use biologically-derived and/or biologically-inspired materials to promote greater host-implant integration and more consistent long-term electrode performance.

## The Foreign Body Response

**FIGURE 1:**
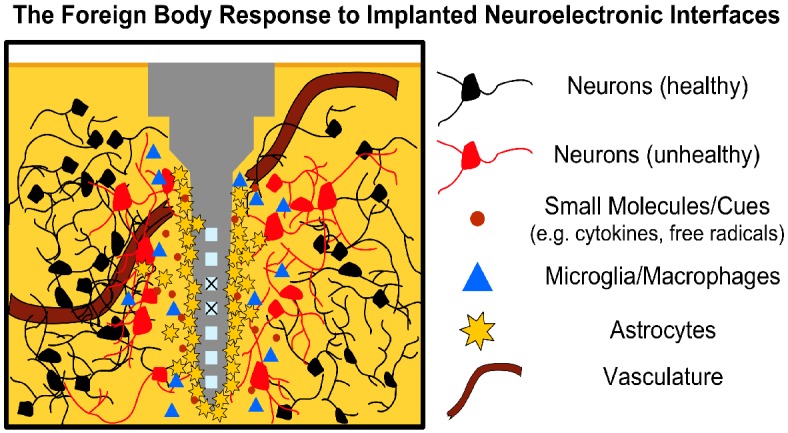
The FBR to Neural Interfaces. Neural interfaces disrupt local tissue triggering an acute immune response wherein local immune cells (microglia, astrocytes) migrate to the injury site and begin secreting pro-inflammatory factors (e.g., cytokines, nitric oxide, free radicals). Astrocytes begin forming a glial scar around the implant over the course of a few weeks, increasing tissue impedance, while disruption of the BBB allows blood-borne macrophages to infiltrate the area. Prolonged inflammation leads to neuronal degeneration and may corrode the implant (“X” over active sites), further limiting electrode function. Note that although the microelectrode depicted represents a silicon shank (i.e., Michigan-style electrode), the concept applies similarly to other microelectrode types, such as the Blackrock Utah array.

The FBR is a neuroinflammatory reaction to the disruption of healthy tissue and continued presence of a foreign body in the brain (Figure 1) (Polikov et al., 2005; Harris and Tyler, 2013). It begins at implantation, which itself causes physical trauma as the electrode(s) displaces and damages vasculature and the blood–brain barrier (BBB), cells, and extracellular matrix (ECM) on its path to the intended target (Sommakia et al., 2014). Subsequently, blood-borne macrophages and other foreign plasma components enter the area, while local microglia and astrocytes begin to transition from resting to active/phagocytic phenotypes as part of the brain’s normal response to injury (Polikov et al., 2005; Harris and Tyler, 2013). Microglia have been observed responding as quickly as 30 min post-delivery, extending processes toward the implant and transitioning to an active phenotype over the course of a few hours (Neumann et al., 2009; Kozai et al., 2012). Activated microglia and macrophages release a battery of pro-inflammatory chemokines, cytokines, and other factors into the damaged area (e.g., tumor necrosis factor, interleukin-1, nitric oxide); while these factors are associated with remodeling tissue and degrading foreign materials following injury, they also cause neurodegeneration (Neumann et al., 2009; Harris and Tyler, 2013).

In the weeks following implantation, a fibrous envelope of reactive astrocytes, connective tissue and ECM, commonly referred to as the glial scar, gradually forms around the device, insulating the foreign body from the surrounding brain tissue (Harris and Tyler, 2013; Sridharan et al., 2013). This glial scar has been a hallmark of neural interfaces in the brain, with experimental strategies often using the extent or thickness of the scar as a measurement for the effectiveness of mitigating the FBR (Sridharan et al., 2013). Growth-inhibiting molecules, such as chondroitin sulfate proteoglycans, also populate the glial scar, further reducing the potential for neuronal growth and recovery in the implant site (Zhong and Bellamkonda, 2007). The presence of the implant in the brain generally causes a sustained inflammatory response, with both astrocytes and microglia remaining in the area in a pro-inflammatory state in an attempt to eliminate the foreign body (Polikov et al., 2005; Harris and Tyler, 2013; Woeppel et al., 2017). The continued release of neurotoxic factors from the active microglia/astrocytes is detrimental to local neurons, with many studies reporting a decrease in the neuronal density surrounding the implant (Polikov et al., 2005).

To date, the mechanisms of the FBR are still not completely understood. As such, the relationship between various elements of the FBR and failure modes of chronically-implanted neuroelectronic interfaces is still an area of active study (Polikov et al., 2005; Winslow and Tresco, 2010; Jorfi et al., 2015; Sahyouni et al., 2017; Woeppel et al., 2017). What is known is that the introduction of any such interface to the CNS induces multi-phase tissue remodeling that results in glial scarring, prolonged BBB disruption, and the persistent presence of pro-inflammatory elements that collectively form an adverse microenvironment for neural interfacing (Figure 1). This microenvironment poses several active challenges to both the device and the neurons of interest (Groothuis et al., 2014; Nolta et al., 2015; Woeppel et al., 2017). The biostability of the former is continually challenged by reactive oxygen species, which corrode active electrode sites and gradually degrade insulating layers and device interconnects (Groothuis et al., 2014; Nolta et al., 2015; Woeppel et al., 2017). Other failure modes, such as mechanical failure and micromotion-induced shear as the brain shifts, may further drive inflammation in a positive feedback manner (Polikov et al., 2005; Jorfi et al., 2015). As noted above, local astrocytes around the implant eventually form the glial scar, which physically separates the device from the neurons of interest and increases the electrical impedance of local tissue. Moreover, the continued presence of reactive immune cells, cytokines, and other inflammatory factors at recording/stimulation sites induce neuronal death and/or prevent the restoration of healthy neural tissue (McConnell et al., 2009; Winslow and Tresco, 2010; Fattahi et al., 2014; Shoffstall and Capadona, 2018). Disruption of the BBB has also been implicated as a significant link between the FBR and the decline in interface performance over chronic periods, with “leakiness” of the BBB allowing peripheral immune cells to enter the brain parenchyma and accumulate in the lesion to exacerbate neurotoxic effects at longer timepoints (Saxena et al., 2013; Woeppel et al., 2017; Bennett et al., 2018).

## The Challenge of Biological Compliance

The complex and multi-faceted challenge of designing long-acting neural interfaces has engendered an ongoing, cross-disciplinary mission to improve their biological compliance, defined as their ability to induce favorable – or at least not disrupt – cell- and tissue-level interactions. These strategies span mechanical design, materials science (across nano to macro scales), immunology, neurobiology, electrical engineering, and tissue engineering, among others; a subset of the milestones in the field are summarized below and are referenced in more in-depth analyses (Schmidt and Leach, 2003; Cullen et al., 2011; Jorfi et al., 2015; Sahyouni et al., 2017).

## Electrode Material Properties and Geometry

It has been shown that reducing electrode size minimizes the trauma of insertion and can reduce the severity of the glial scar in chronic implants (Stice et al., 2007; Karumbaiah et al., 2013). Similarly, electrodes with open-faced geometries (e.g., lattices, meshes) minimize the total surface area of the interface, while permitting diffusion throughout the area, with rodent models showing reduced microglial reactivity and higher neuronal density out to at least 1 month (Seymour and Kipke, 2007; Sommakia et al., 2014). Further, the lattice topography has been shown to influence not only the extent but the distribution of scarring around the implant, potentially leveraging it to improve contact with brain tissue (Schendel et al., 2014a,b). One such mesh electrode comprised of flexible nanowire transistors assembled in a flexible, lightweight sheet was able to record both single units and field potentials in mice for several months; histological assays showed both a lack of glial proliferation and neuronal attrition surrounding the implant for at least 1 year, suggesting that the unique geometry leaves the host tissue relatively unperturbed (Hong et al., 2018). Notably, this mesh leverages conductive ink and computer-controlled stereotactic injection to enable connection to standard electrophysiological equipment and reproducible targeting of brain regions, respectively (Hong et al., 2018).

In addition to geometric changes, reducing the stiffness of the implant minimizes the mechanical discrepancy between the device and host tissue; the use of polymers or “mechanically adaptive” materials which are stiff enough for insertion but soften upon implantation has demonstrated significant reductions in long-term neuroinflammation, immune cell activation, and neurodegeneration (Harris et al., 2011; Jeon et al., 2014; Nguyen et al., 2014; Sridharan et al., 2015; Lecomte et al., 2018). Materials science approaches to biological compliance include the patterning of nanoscale topography to better integrate with features of local tissue, increasing the effective surface area of the implant, and development of electrodes with new materials such as carbon nanotubes, which have demonstrated favorable electrochemical properties and reduced immunoreactivity compared to traditional probes (Webster et al., 2004; Saito et al., 2009; Heim et al., 2012; Vitale et al., 2015; Jalili et al., 2017; Scaini and Ballerini, 2018). Manipulating the surface chemistry of implanted materials may also improve biological compliance; certain hydrophilic or negatively-charged functional groups such as -COOH may reduce glial scarring, depending on their affinity for protein binding or cell membranes (Christo et al., 2015; Yu et al., 2015). Increasing the surface permeability of implant coatings to serve as “diffusion sinks” for pro-inflammatory molecules has also reduced immunoreactivity around the electrode (Skousen et al., 2015).

## Bioactive Electrodes

**FIGURE 2:**
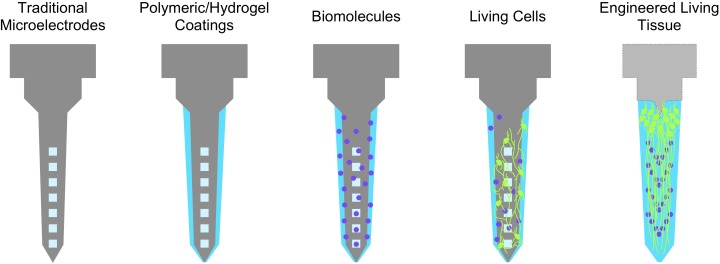
The spectrum of bioactive neuroelectronic interfaces. Non-organic electrodes may be coated with soft biomaterials of hydrogels for better mechanical parity, which may in turn be functionalized with various biological molecules (e.g., soluble factors, ECM proteins) to transiently reduce inflammatory phenomena or provide support for neuronal growth and attachment. The incorporation of living cells may further provide sustained trophic support and a more biofidelic interface, while engineered tissue (i.e., with an organized structure, as an electrode coating or a self-contained implant) may be the closest representation of “self” attainable with respect to native tissue.

Improving biological compliance can be described as minimizing the degree of discrepancy between the self (host tissue) and not-self (foreign implants). In this context, the more closely a given interface approximates properties of biological tissue (“self”), the higher the chances of chronic stability and integration with the tissue of interest. This principle motivates the development of bioactive neural interfaces, which attempt to improve biological compliance through the elicitation, suppression, or otherwise modulation of specific biological phenomena. Broadly, this class of interfaces is designed to incorporate, mimic, or draw inspiration from pre-existing, biologically-derived materials; candidate materials are selected for their effects on cellular or physiological processes (e.g., attenuation of the immune response, promotion of neuron attachment, and growth) (Shoffstall and Capadona, 2018). These strategies are designed to improve the prospects of long-term function while reducing the complications from the presence of a foreign body. Bioactive interfaces may be visualized as a spectrum ranging from completely inorganic, non-biological devices to living engineered constructs (Figure 2). They may incorporate proteins or drugs that downregulate specific mechanisms of immunoreactivity (e.g., microglial activation), inflammation (e.g., cytokine release, glial scar formation), promote neuronal attachment or neurite outgrowth, recruit endogenous neuroprotective mechanisms, or present *de novo* cells or tissue to replace lost neurons and supporting architecture (Shain et al., 2003; Zhong and Bellamkonda, 2007; Purcell et al., 2009; Cullen et al., 2011; Taub et al., 2012). Most current bioactive interfaces take the form of traditional inorganic electrode materials (e.g., platinum, tungsten, silicon) surrounded by coatings that contain or are comprised of biomolecules as described below (Aregueta-Robles et al., 2014; Szostak et al., 2017). These biomaterial coatings are generally several orders of magnitude softer than the enclosed material to provide better mechanical parity with the brain; common coatings include silk, polyimide, and parylene, and various hydrogels or synthetic polymers (Zhong and Bellamkonda, 2008; Green et al., 2009; Chen and Allen, 2012; Balint et al., 2014; Mario Cheong et al., 2014).

Anti-inflammatory agents such as the steroid dexamethasone or α-MSH have been incorporated into electrode coatings to limit the production of inflammatory cytokines and other glial by-products; these approaches generally result in reduced glial scarring around neural implants in animal models, although they are limited by the release and eventual depletion of the agent in use (Zhong and Bellamkonda, 2005; Abidian and Martin, 2009; Kim et al., 2010; Aregueta-Robles et al., 2014; Boehler et al., 2017). Other biological molecules associated with neuronal attachment, structural support, or migration (e.g., laminin, L1, collagen), may be entrapped or immobilized through covalent bonding to both natural and synthetic polymer coatings to present a more attractive surface for neurons; a common strategy is the doping of conductive polymers (of which the most prevalent for neural interfacing are PEDOT and polypyrrole) with biomolecules to improve their biocompatibility (Green et al., 2008; Azemi et al., 2010, 2011; Bendrea et al., 2011; Chen and Allen, 2012; Hardy et al., 2013; Balint et al., 2014; Sommakia et al., 2014; Green and Abidian, 2015). A wealth of *in vitro* studies have demonstrated neural cell survival and process outgrowth on substrates functionalized with growth factors (i.e., NGF, NT3, BDNF) and ECM proteins (laminin, collagen); *in vivo*, histological analyses of these bioactive coatings reveal attenuation of the glial response 4–8 weeks post-implant, with some studies reporting higher local neuronal survival compared to uncoated electrodes (He and Bellamkonda, 2005; Green et al., 2008; Thompson et al., 2010; Azemi et al., 2011; Liu et al., 2011; Chen and Allen, 2012; Fattahi et al., 2014; Mario Cheong et al., 2014; Sommakia et al., 2014; Kozai et al., 2015; Shen et al., 2018; Shoffstall and Capadona, 2018; Vitale et al., 2018). However, while bioactive materials provide greater biocompatibility to these devices, ongoing challenges for these strategies include limited duration of effect as biomolecules diffuse away from the implant (with no mechanism for replenishment), are removed by local microglia or competitive binding, or undergo undesired modification (e.g., pH-driven conformational changes), which collectively result in poor translation of results from *in vitro* assays to *in vivo* implants (Aregueta-Robles et al., 2014; Kozai et al., 2015). Further, the benefits borne out by histological studies vary – e.g., diminished glial scarring with no evidence of improved neuronal survival – and have largely not yet been tied to improved functional outcomes (He et al., 2006; Aregueta-Robles et al., 2014; Jorfi et al., 2015; Michelson et al., 2018). Validating the clinical potential of these bioactive implants requires meeting the benchmarks set by current interfaces in both non-human primates and humans. For instance, despite well-known limitations in their long-term biostability, inorganic electrodes such as the Blackrock microelectrode array provide the clinical performance foundation and have been the source of significant milestones in neuroprosthesis research (Hochberg et al., 2012; Klaes et al., 2014; Gilja et al., 2015). As such, these existing devices set the standard by which bioactive interfaces will be evaluated as they evolve from a growing body of promising results *in vitro*, to improved performance and reproducibility in model systems *in vivo*, and, potentially, in clinical applications.

## Electrodes Decorated With Living Cells or Tissue

There are clear, data-driven benefits to engineering neural interfaces as bioactive devices. Increasing similarities between the implant and tissue create further opportunities for greater neuronal contact, reduced chronic inflammation, and more stable long-term function. However, bioactive interfaces to date are introduced to the brain with fixed quantities of biomolecules, which may become depleted or removed due to natural biological processes. Toward this end, new research efforts have begun to explore whether living cells may act as active elements of a neuroelectronic interface, potentially matching the dynamic nature of brain tissue (Cullen et al., 2011; Shoffstall and Capadona, 2018). Cell-based interfaces may leverage the self-driven machinery of living cells to actively produce neuroprotective factors while presenting a material that mimics the “self” enough to downregulate chronic inflammation, although these potential advantages must be developed and validated *in vivo*. One such study coated microelectrodes with a fibrin hydrogel containing primary astrocytes and neurons; although the fibrin was resorbed within one week following implant in rat cortex, astrocyte reactivity was diminished out to at least 30 days post-implant compared to bare electrodes (De Faveri et al., 2014). Further, the inclusion of a cell layer did not significantly affect recordings from the electrodes themselves, although effects on the survival of host neurons were not reported (De Faveri et al., 2014). Other studies have trapped live neurons within conductive polymers; although residual monomers have proven cytotoxic and negatively impacted cell survival beyond a few days, the polymerized network may serve as a three-dimensional, electrically functional scaffold (Richardson-Burns et al., 2007a). A similar approach polymerized *in vivo* the conductive polymer PEDOT within the brain, resulting in a network of conductive filaments surrounding neurons and tracking white matter (Richardson-Burns et al., 2007b). Although the network was electrochemically validated as a functional electrode, further work is required to determine effects on cellular viability, network behavior, and whether the distribution of the polymer can be controlled for precise stimulation or recording. Green et al. (2013) demonstrated a multi-layer biohybrid interface consisting of platinum electrodes, conductive polymer-hydrogel blend, and PC12 cells within a biodegradable hydrogel layer; cells survived out to 12 days *in vitro* and extended neuritic processes upon the addition of NGF. Potential future development would interrogate whether such embedded neural cells are capable of synaptogenesis, forming a functional neuronal layer around the electrode (Green et al., 2013; Aregueta-Robles et al., 2014).

In addition to dissociated or embedded cells, the combination of living tissue and neuroelectronics may further leverage the functional benefits of the ECM surrounding the neurons, including the presence of signaling molecules, structural support, tissue-level organization, and the dynamic remodeling of the matrix to facilitate growth or stabilize neuronal networks (Cullen et al., 2011). Further, the introduction of support cells (e.g., glia) in pro-regenerative states may provide sufficient cues to prevent or ameliorate the neurodegenerative outcomes present in the chronic inflammatory response (Aregueta-Robles et al., 2014). The first such interface, a “neurotrophic electrode,” was reported by Dr. Philip Kennedy in a 1989 paper, where a glass pipette electrode was seeded with a piece of sciatic nerve and implanted into rat and later monkey cortex (Kennedy, 1989; Kennedy et al., 1992). Neurites grew into the tip, while the extent of growth correlated with tip diameter; recordings from these early living, biohybrid devices lasted over a year (Kennedy et al., 1992). Notably, a solution of NGF in the same pipette had the opposite effect, with a cystic cavity forming around the implant; these results suggest that the benefits of soluble factors may be further improved with the innate, dynamic regulation present in the nerve explant and/or host tissue (Kennedy, 1989). Thus, leveraging robust and multi-faceted biological mechanisms from living tissue may enhance electrode performance in the brain. However, as a relatively new evolution in the neuroengineering field, the advantages of cell- and tissue-seeded electrodes are still largely under active exploration in *in vitro* assays and rodent models (Jorfi et al., 2015; Shoffstall and Capadona, 2018). As with bioactive material-based interfaces, translating these presumed advantages into better interface performance and functional outcomes requires further validation.

## Micro-Tissue Engineered “Living Electrodes”

**FIGURE 3:**
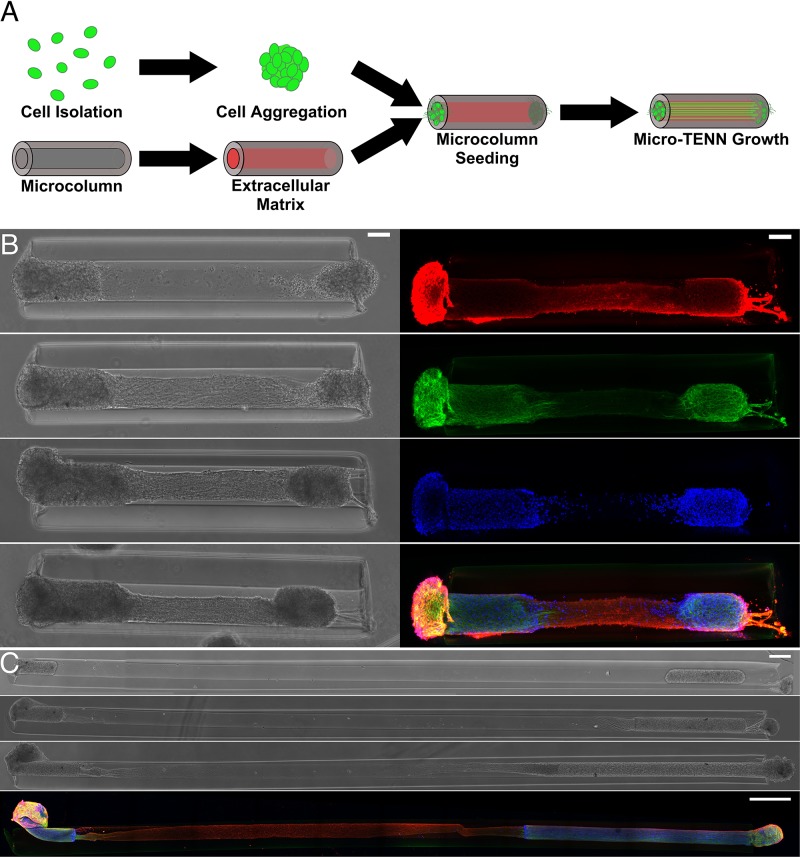
Micro-TENNs as living, 3D constructs. **(A)** Neurons are isolated to form spheroid aggregates that are placed within hydrogel microcolumns filled with ECM. Micro-TENNs are then grown *in vitro*. Either 1 aggregate or 2 aggregates to form either unidirectional or bidirectional micro-TENNs, respectively. **(B)** Left: phase microscopy images of a 1.5 mm bidirectional micro-TENN at 1, 3, 4, and 7 days *in vitro* (DIV). Right: confocal reconstructions of the same micro-TENN stained to identify axons (Tuj-1; red), cell soma/dendrites (MAP-2; green), and cell nuclei (Hoechst; blue). Scale bars: 100 μm. **(C)** Phase microscopy images of a 9 mm bidirectional micro-TENN at 1, 3, and 7 DIV. The bottom image is a confocal reconstruction of this micro-TENN with the same labeling as in **(B)**. Scale bars: 500 μm. Adapted with permission of IOP Publishing from Dhobale et al. (2018).

A recent potential neuroelectronic interface strategy developed by our research group involves the engineering of self-contained, functional neural tissue preformed *in vitro* that may be applied toward a myriad of regenerative and neuroprosthetic functions. These micro-tissue engineered neural networks (micro-TENNs) consist of microscopic hydrogel cylinders (micro-columns) with ECM optimized for axonal growth within the central lumen (Struzyna et al., 2015; Adewole et al., 2018; Serruya et al., 2018). Spherical aggregates of primary neurons placed at the micro-column terminals extend neurites through the ECM lumen over time, forming a three-dimensional network of aligned axonal tracts spanning one (unidirectional) or two (bidirectional) neuronal populations (Figure 3). Following network formation, these constructs may be precisely implanted in the brain to enable synaptic integration with target regions. Micro-TENNs were originally developed to replace long axonal brain pathways that are often compromised or lost due to traumatic brain injury or neurodegenerative disease, with an anatomically-inspired distribution of discrete cell body aggregates and axon tracts designed to recreate the segregation of gray and white matter in the mammalian brain (Cullen et al., 2012; Struzyna et al., 2015, 2017).

As engineered micro-tissue, micro-TENNs are unique in that their design enables a high level of control over their mechanical, material, and biological properties, while their structure mimics the natural network-level architecture of the brain. The hydrogel micro-column provides a structure to coax the neuronal and axonal growth into the desired architecture, and may be made from a range of biomaterials with varying porosity, stiffness, degradation kinetics, or similar properties as needed. The ECM in the lumen is tailored to support neuronal growth and maturation, and may be modified to contain additional structural proteins and/or chemotactic cues for axonal support and guidance.

After the desired growth and maturation are achieved, the micro-column allows for manipulation of the preformed neural network as a single unit and serves as a protective encasement to chaperone microinjection into the brain. Within the context of the FBR, the micro-column and luminal ECM also protect the neurons and axonal tracts against the potentially inflammatory post-injection microenvironment. The smallest micro-TENNs to date are only ∼320 μm in diameter, permitting minimally-invasive delivery to the brain; simultaneously, they may be made to different lengths (from 100s of microns to centimeter-scale constructs) to span large deficits or tap into deeper brain structures (Struzyna et al., 2017; Adewole et al., 2018).

In addition, the scalable bio-fabrication process is amenable to isolating precise neuronal subtypes to maintain control of the effects of micro-TENN synaptic inputs on host circuitry. To date, micro-TENNs have been fabricated using cerebral cortical neurons (e.g., mixed glutamatergic and GABAergic), dorsal root ganglia neurons (e.g., sensory), ventral mesencephalic neurons (e.g., dopaminergic), and medial ganglionic eminence neurons (e.g., GABAergic), amongst other neuronal subtypes, from a range of species including rodent, porcine, and human sources. Moreover, the process of engineering neuronal aggregates creates the opportunity for viral transduction based on neuronal phenotype and protein expression profiles. For example, functionalization of the micro-TENNs with optogenetic actuators (e.g., channelrhodopsin) and optical reporters such as GCaMP allow for light-driven control and monitoring of the constructs for *in vitro* or *in vivo* applications (Struzyna et al., 2017; Adewole et al., 2018). Thus, axon-based living electrodes provide an ability for natural, synaptic-based excitation, inhibition, and/or modulation of host circuitry under optical control.

**FIGURE 4:**
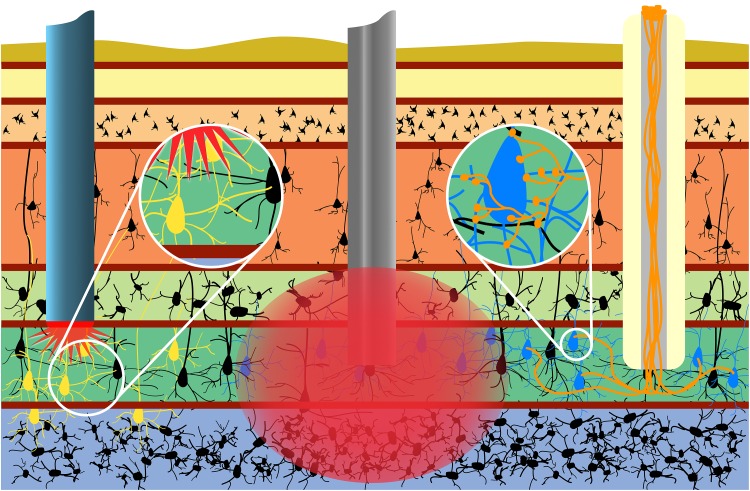
Micro-TENNs applied to neural interface as living electrodes. Living electrodes (right) may provide engineered axonal tracts in a controlled 3D cytoarchitecture to relay signals to/from the brain. Designing constructs to synapse with specific neuronal subtypes (blue neurons) may offer higher specificity than conventional electrodes (middle), which stimulate/record from a 3D volume (red), or than optical stimulation methods (left), wherein the introduction of virus to target neurons (yellow) may spread to non-target regions (e.g., yellow neurons across multiple cortical layers). Reprinted with permission of John Wiley and Sons from Serruya et al. (2018).

Indeed, within the context of neuroelectronic interfaces, micro-TENNs may serve as a living information relay (or “living electrode”) between deep targets in the brain and an apparatus on the brain surface (Figure 4). In this paradigm, these living electrodes may be stereotactically microinjected such that the deep axon tracts may form synapses with targeted areas of the brain while the neuronal cell bodies remain at the brain surface, allowing for signal propagation along the internal axonal tracts either from the brain surface to the host tissue or vice versa (Serruya et al., 2018). An appropriate electrical (e.g., micro-ECOG) or optical (e.g., LED array) apparatus may then be mounted on or directly above the brain surface, providing computer-controlled modulation or monitoring of the neural targets through stimulation or recording of the dorsal micro-TENN aggregate, respectively.

**FIGURE 5:**
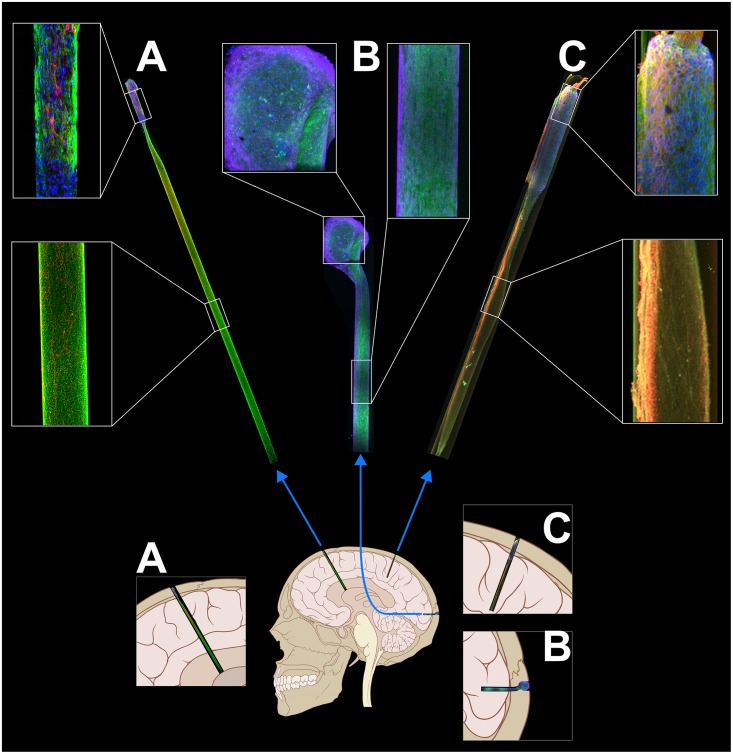
Tissue-engineered living electrodes may be tailored to various applications for neuromodulation. **(A)** Dopaminergic constructs may provide “living deep brain stimulation” for Parkinson’s disease through restoration of dopaminergic inputs to the striatum. **(B)** Living electrodes with GABAergic neurons may inhibit epileptic brain activity. **(C)** Glutamatergic constructs could serve as a means to relay sensory feedback, e.g., from prosthetic limbs. Adapted with permission of John Wiley and Sons from Serruya et al. (2018).

One significant advantage of this approach is that the non-organic stimulation or recording device is isolated to the brain surface or outside the skull entirely, while only the living electrode (comprised solely of soft biomaterials, ECM, and neurons) penetrates the parenchyma (Serruya et al., 2018). Moreover, the creation of optogenetically-active constructs *in vitro* prior to *in vivo* delivery obviates the need to inject viral components directly into the brain (as is the case with conventional optogenetic approaches). Overall, the presentation of exclusively biocompatible materials may curtail the chronic inflammatory response experienced by non-organic implants, while the hydrogel micro-column protecting the axons may be tuned to degrade at an optimal rate such that the axons are gradually introduced to the microenvironment as the tissue recovers. Additionally, as an alternative to microinjection, living electrodes may be encased in a secondary biomaterial sheath that is stiff enough to penetrate the brain and softens when hydrated, eliminating the need for needle delivery and further minimizing the severity of the tissue disruption upon initial delivery (Harris et al., 2016). Functional studies *in vitro* have shown that these constructs are capable of signal propagation through both electrical and optical stimulation, while implants in a rat model have survived out to at least 1 month with evidence of synaptogenesis and confirmation of transplant activity via intravital calcium imaging (Adewole et al., 2018; Struzyna et al., 2018). Finally, control over the neuronal subtype and protein expression prior to implant as described above may provide the opportunity for precise neuromodulation or therapeutic intervention, such as a computer-controlled “living DBS” electrode made using dopaminergic neurons for controlled dopamine replacement/inputs into the striatum for treatment of Parkinson’s disease (Figure 5). Similarly, computer-controlled inhibitory living electrodes may be applied to seizure foci in cases of intractable epilepsy, where detection of early epileptiform activity triggers release of copious GABA to extinguish activity in hyperexcitable circuitry (Figure 5).

While this living electrode strategy is promising and addresses a number of major challenges in the field, there are a several non-trivial challenges to translation that underscore the increased complexity of engineering a biohybrid interface for reproducible function. For instance, as the living electrode concept is based on synapse formation between the implant and brain, the effective stimulation/recording density is dictated primarily by the extent and specificity of synaptogenesis. As such, one significant translational challenge is control over the degree and targeting of synaptogenesis upon implantation. Constructs may be seeded with neurons that preferentially synapse with specific subtypes for more targeted interfacing, although the proportion of desired to aberrant connections is an ongoing area of investigation. Further, migration of living electrode neurons from the construct over time has been observed in early-stage implants, potentially necessitating a mesh or similar barrier to prevent neuronal migration away from the target stimulation/recording site at the brain surface. In the case of bidirectional living electrodes for recording, activity from host neurons must be conveyed across at least two synapses, making them potentially useful for recording local fields but likely hindering the ability to isolate single neurons from the output activity at the brain surface. Computer models of living electrodes may provide predictions of synaptogenesis and signal propagation to better inform design choices and interpret neuronal activity (Dhobale et al., 2018). Clinical bio-fabrication will also be a significant challenge for translation, including starting biomass, quality assurance, and safety monitoring. Here, the use of autologous, stem cell derived neurons would mitigate the need for immune suppression, although personalized living electrodes would be more expensive to build and more challenging to validate than allogeneic living electrodes from a standardized neuronal source. Finally, the input/output behavior of the living electrode under external control must be characterized to (1) compare the living electrode behavior to current clinical benchmarks for neuronal interfacing, and (2) determine the best method of external control at the brain surface. The choice of electrical (e.g., μECOG) or optical interfacing (e.g., LED array) will also likely need further optimization depending on the target application.

## Summary

Despite decades of significant effort, to date there is no single ideal neuroelectronic interface for long-term applications. While the definition of an ideal set of properties for a given interface is determined by the intended application, the clinical viability of these technologies is largely determined by their ability to function stably and predictably over long-term periods, which may, for several applications, span the course of the user’s life. Current interfaces are limited by the multi-phasic FBR, a series of prolonged inflammatory processes that lead to neuronal attrition at the implant site and inhibit the chronic utility of recording or stimulation electrodes. Years of natural selection have provided a vast library of mechanisms for directing neuronal growth, migration, and immunoreactivity; a common design feature of bioactive interfaces is the recruitment or partial recreation of these systems to influence local biological activity for better integration. Currently, bioactive interfaces largely use a combination of minimally invasive, soft material coatings, soluble factors and other biomolecules to limit the implant footprint, curb inflammation, and promote neuronal survival, although sustaining this bioactivity over long periods of time remains a significant design challenge. As such, it is necessary that the next generation of implantable, bioactive interfaces maintain a microenvironment that enables chronically stable performance, potentially through the introduction of living cells or tissue to further minimize the disparity between the implant and host brain. Ultimately, fully biological interfaces, such as living electrodes, may allow for a seamless integration with the host circuitry for controlled neuromodulation, feedback, and, ideally, functional restoration.

## Author Contributions

DKC outlined, edited drafts, and finalized the manuscript. DA performed the literature search, wrote the initial draft, made revisions, and prepared all of the figures. JW and MS made additions and edits to the manuscript.

## Conflict of Interest Statement

DC is a scientific co-founder of INNERVACE, LLC, and Axonova Medical, LLC, which are University of Pennsylvania spin-out companies focused on translation of advanced regenerative therapies to treat nervous system disorders. The remaining authors declare that the research was conducted in the absence of any commercial or financial relationships that could be construed as a potential conflict of interest.
